# Evolution of BCR/ABL Gene Mutation in CML Is Time Dependent and Dependent on the Pressure Exerted by Tyrosine Kinase Inhibitor

**DOI:** 10.1371/journal.pone.0114828

**Published:** 2015-01-28

**Authors:** Shantashri Vaidya, Babu Rao Vundinti, Chandrakala Shanmukhaiah, Prantar Chakrabarti, Kanjaksha Ghosh

**Affiliations:** 1 Department of Cytogenetics, National Institute of Immunohaematology, 13^th^ Floor, New Multistoried Building, KEM Hospital Campus, Parel, Mumbai, 400012, India; 2 Department of Haematology, 10^th^ Floor, New Multistoried Building, KEM Hospital, Parel, Mumbai, 400012, India; 3 Institute of Haematology and Transfusion Medicine, Kolkata, West Bengal, 700073, India; Hungarian Academy of Sciences, HUNGARY

## Abstract

**Background:**

Mutations in the ABL kinase domain and SH3-SH2 domain of the BCR/ABL gene and amplification of the Philadelphia chromosome are the two important BCR/ABL dependent mechanisms of imatinib resistance. Here, we intended to study the role played by TKI, imatinib, in selection of gene mutations and development of chromosomal abnormalities in Indian CML patients.

**Methods:**

Direct sequencing methodology was employed to detect mutations and conventional cytogenetics was done to identify Philadelphia duplication.

**Results:**

Among the different mechanisms of imatinib resistance, kinase domain mutations (39%) of the BCR/ABL gene were seen to be more prevalent, followed by mutations in the SH3-SH2 domain (4%) and then BCR/ABL amplification with the least frequency (1%). The median duration of occurrence of mutation was significantly shorter for patients with front line imatinib than those pre-treated with hydroxyurea. Patients with high Sokal score (p = 0.003) showed significantly higher incidence of mutations, as compared to patients with low/intermediate score. Impact of mutations on the clinical outcome in AP and BC was observed to be insignificant. Of the 94 imatinib resistant patients, only 1 patient exhibited duplication of Philadelphia chromosome, suggesting a less frequent occurrence of this abnormality in Indian CML patients.

**Conclusion:**

Close monitoring at regular intervals and proper analysis of the disease resistance would facilitate early detection of resistance and thus aid in the selection of the most appropriate therapy.

## Introduction

The molecular basis of chronic myeloid leukemia led to the development of a target directed anti-cancer compound, 2-phenylaminopyrimidine (formerly CGP57148, now imatinib mesylate). Although a vast majority of patients with CML respond to the tyrosine kinase inhibitor (TKI) imatinib mesylate (IM), resistance might occur de novo or during treatment [1]. This resistance to TKIs has attracted the attention of many researchers to understand the different mechanisms responsible for the resistance and disease progression. Previous studies have reported that approximately 20% of patients fail therapy with imatinib because of point mutations within the BCR/ABL kinase domain. Sherbenou et al. [2]have confirmed that the mutations outside the kinase domain also contribute to imatinib resistance to a certain extent. In the context of ABL, these domains have an autoinhibitory effect on the kinase activity, and mutations in this region can activate the enzyme. Many studies have also made an important point that the mutations in the kinase domain are selected and not induced by the TKI therapy.

In our study, three different BCR/ABL dependent mechanisms of imatinib resistance; ABL kinase domain mutations, mutations in SH3-SH2 domain of BCR/ABL gene and BCR/ABL amplification were studied in CML patients exhibiting clinical resistance to imatinib. Impact of non-TKI pre-treatment on the subsequent TKI therapy was also studied.

## Materials and Methods

The patients were included in the study after a written informed consent was obtained. The study was approved by the Institutional Ethics Committee for Research on Human Subjects of National Institute of Immunohaematology (ICMR).

### Patients

CML patients (n = 254) treated with imatinib at the Department of Haematology, KEM Hospital, Mumbai, and Institute of Haematology and Transfusion Medicine, Kolkata, India, from March 2008 to Dec 2013 were included in the study. Only patients with good a compliance to the imatinib therapy were included in the study. Good compliance was assured by (i) Extensive counselling (ii) Providing the responsibility of giving medicine to the patient by closest relative (wife where husband was affected, parents where child was affected) (iii) Counting the remaining tablets (iv) Randomly checking the sera for inhibitory activity on BCR/ABL imatinib sensitive cell lines. European LeukeimaNet definitions were considered to categorize patients as imatinib resistant [3].

At the time of resistance, 28 patients were pre-treated with a non-TKI (hydroxyurea) and 66 patients were given front-line imatinib treatment. Of the 94 IM resistant patients, 76 patients were in chronic phase (CP; defined as less than 10% blasts in peripheral blood or bone marrow, less than 20% blasts plus promyelocytes in peripheral blood or bone marrow, less than 20% basophils and no extramedullary involvement except from the liver/spleen), 9 in accelerated phase (AP; defined as less than 20% blasts in peripheral blood or bone marrow, presence of at least 20% basophils in blood) and 9 in blast crisis (BC; defined as more than 20% blasts in blood or bone marrow or presence of extramedullary blast crisis). The imatinib treatment regime for CP patients was 400mg/day and for AP/BC was 600mg/day, except for slight adjustments for patients with myelo-suppression. Characterization of resistance was based on the recommendations by European LeukemiaNet 2009 [3] for management of CML patients: lack of complete haematologic response (CHR) or Ph+ >95% by 3 months, Ph+ >35% or BCR-ABL^IS^>10% by 6 months, BCR-ABL^IS^>1% or Ph+ >0% by 12 months, loss of complete haematologic/ cytogenetic/ molecular response at any time during the treatment or progression from CP to AP/BC. Patients not achieving the desired milestones (CHR by 3 months or Partial Cytogenetic Response (PCyR) by 6 months or Major Molecular Response(MMR) by 12 months) were categorised as primary resistant and patients losing the previously achieved haematologic or cytogenetic response or patients exhibiting a consecutive 1 log increase in transcript level after achieving a MMR were categorised as secondary resistant patients.

### Cytogenetic study

Conventional cytogenetic analysis was performed according to the standard procedure. The chromosomal preparations were subjected to GTG-banding. At least 30 well spread and good banded metaphases were analysed and karyotyped according to the International System for Human Cytogenetic Nomenclature 2009 [4].

### Fluorescence in situ hybridization (FISH)

The FISH probes used in the study are dual colour locus specific probes (Vysis) for detection of BCR/ABL fusion gene. FISH was carried out by denaturing the probe and metaphase and the procedure was carried out according to manufacturer’s instructions.

### DNA and RNA extraction and cDNA conversion

DNA isolation was done from peripheral blood or bone marrow samples using QIAamp DNA blood mini kit (Qiagen, Limburg, Netherlands) according to the manufacturer’s instructions. The isolated DNA was stored at -20°C. To minimize RNA degradation, RNA isolation was done from fresh blood sample within 20mins of collection. Total RNA was extracted using QIAamp RNA blood mini kit (Qiagen, Limburg, Netherlands) according to the manufacturer’s instructions and stored at -80°C. Total RNA was reverse transcribed to first strand cDNA using First strand cDNA synthesis kit (Thermo Scientific, Pennsylvania, USA).

### Direct sequencing

**PCR conditions**: cDNA samples were amplified using respective oligonucleotide primers (Sigma, Bangalore, India)in a 96 well thermal cycler (Applied Biosystems, Veriti, Carlsbad, California). Briefly, in a reaction volume of 25μl, 1X Long PCR buffer (complete) and 0.5U Long PCR Enzyme Mix (Thermo Scientific, Pennsylvania, USA);2 mM MgCl2; 0.75mM each dATP, dCTP, dGTP, and dTTP (Thermo Scientific, Pennsylvania, USA); 0.3 μM primers each and 2μl of cDNA were added.

**ABL kinase domain**: The complete ABL kinase domain of the BCR/ABL gene was analysed using semi-nested PCR followed by direct sequencing using previously published primers [5]. First-stage PCR (annealing 60°C): Forward primer BCRF (5′- TGACCAACTCGTGTGTGAAACTC) and reverse primer ABLKinaseR (5′-TCCACTTCGTCTGAGATACTGGATT). A second-stage PCR (annealing 62°C):: Forward primer ABLkinaseF (5′-CGCAACAAGCCCACTGTCT) and reverse primer ABLkinaseR.

**Regulatory domain**: The regulatory region consisting of neighboring linker, SH3, SH2 and cap region were analysed as described earlier [6]. First-stage PCR (annealing 60°C):: Forward primer BCRF (5′- TGACCAACTCGTGTGTGAAACTC) and reverse primer ABLKinaseR (5′-TCCACTTCGTCTGAGATACTGGATT). A second-stage PCR (annealing 62°C):: Forward primer BCRF and reverse primer ABLkinaseR1 (5′- CTGTCATCAACCTGCTCA GG).

Sequencing was done in forward and reverse direction using the Big Dye Terminator chemistry of ABI Prism 310 sequencer (Applied Biosystems, Foster City, USA) by BigDye terminator v3.1 cycle sequencing reaction. Sequences were compared with the native BCR/ABL gene sequence (NCBI reference sequence 005157.4).

### ASO-PCR for T315I

The ASO-PCR to detect T315I mutation was performed using following sets of primers [7]. T315I mutation, F315C: 5′- GCCCCCGTTCTATATCATCAC 3 or F315T: 5′-CCCGTT CTATATCATCAT 3 and R1: 5′-GGA TGAAGT TTT TCT TCT CCA G 3 (annealing at 64°C; 158-bp PCR product). In a reaction volume of 25μl, 1X PCR buffer (complete) and 0.5U Taq (Banglore Genei, India), 1.5 mM MgCl2; 0.25mM each dATP, dCTP, dGTP, and dTTP (Thermo Scientific, Pennsylvania, USA); 0.3 μM primers each and 1μl of DNA were added.

### Statistical analysis

Statistical analysis was carried out using Graph Pad In Stat 2 software (Graph Pad Software Inc., La Jolla, CA, U.S.A.). A p-value ≤0.05 was considered statistically significant. Event free survival (EFS) was calculated from the start of the TKI treatment until loss of response, progression to AP/BC or TKI switch over. Overall survival (OS) was calculated from the start of the treatment until death or until mutation detection. For overall survival comparison, Kaplan-Meier curves were plotted and compared using log-rank test. PolyPhen 2 software was used to predict the possible impact of an amino acid substitution on the structure of the protein using straight forward physical and comparative considerations.

## Results

In this study, 94 of 254 CML patients exhibited resistance to imatinib. Clinical details of imatinib responders and non-responders are given in Table 1. A higher percentage (90%) of patients had secondary resistance(85 secondary v/s 9 primary).

**Table 1:** Clinical details of imatinib responders and non-responders.

	No. of IM responders	No. of IM resistant patients
**Age**	40 (4–72)	37 (8–75)
**Sex**		
Male	109 (67%)	63 (67%)
Female	51 (32%)	31 (33%)
**Disease Phase at resistance**		
Chronic Phase	Not applicable	76 (80%)
Accelerated Phase	Not applicable	9 (10%)
Blast Crisis	Not applicable	9 (10%)
**Non-TKI pre-treatment**		
Busulfan	0	1
Hydroxyurea	43	27
No	117	66
**Response milestones achieved**		
Haematological response (HR) at 3 months		
Complete HR	154	74
Partial HR	6	7
No HR	0	13
Cytogenetic response (CyR) at 12 months		
Complete	123	41
Partial	37	24
Minor	0	5
Minimal	0	12
None	0	12
Molecular response (MoR) at 18 months		
Complete	133	12
Major	27	40
No	0	42
Expired during the course of treatment	0	5
**Dose of imatinib at disease resistance**		
400mg/day	Not applicable	24
600mg/day	Not applicable	34
800mg/day	Not applicable	27
On HU due to imatinib failure	Not applicable	9

### Frequency of mutations

Mutations in the BCR/ABL gene (KD plus the SH3-SH2 domain) were found in 41 (44%) patients of the 94 imatinib resistant CML patients evaluated. Twenty four different mutations were found in these 41 patients. Of these 24 mutations, 19 were point mutations, whereas 5 were insertion/deletion mutations. Only two patients showed presence of multiple mutations. In this study, 6 novel mutations were identified; 3 point and three insertion/deletion. Details of mutations are given in Table 2. All the mutations were detected only after the commencement of imatinib therapy and none had mutations at diagnosis.

**Table 2:** Spectrum of mutations in the ABL KD and SH3-SH2 domain of BCR/ABL gene according to the disease phase.

	CP	AP	BC	Total	PPhV2 score
**Patients with imatinib resistance**	76	9	9	94	
**Patients with mutations in BCR/ABL gene**	29	5	7	41	
**Patients with mutations in kinase domain**				37	
*P loop mutations*				16	
M244V	1	0	0		0.907
K247E	1	0	0		0.814
G250E	2	1	1		0.999
Y253F	1	1	0		1
Y253H	0	2	4		1
E255V	1	0	0		0.951
del L248-K274*	1	0	0		
*Gatekeeper*				5	
T315I	3	0	1		1
F317L	1	0	0		1
*Catalytic domain*				11	
M351T	1	0	1		0.927
M351V	1	0	0		0.927
E355G	1	0	0		0.986
Ins GAA at 357^th^ amino acid*	1	0	0		
F359V	1	0	0		0.989
F359I	1	0	0		0.989
35bp ins of intron 8 at exon 8–9 junction	4	0	0		
*Activation domain*				3	
L387M	1	1	0		0.969
H396R	1	0	0		0.983
*Patients with mutations in SH3-SH2 domain*				4	
K84E*	1	0	0		0.973
Y167H*	1	0	0		0.677
del Exon 4	2	0	0		
*Multiple mutations*				2	
G250E-E255K	1	0	0		
Ins CAGG* at 303^th^ amino acid and F493L	1				

* Novel mutations detected

### Mutations in the BCR/ABL gene and disease phase

Mutations were detected in 29 of 76 imatinib resistant CML patients in CP, 5 of 9 patients in AP and 7 of 9 patients in BC. The occurrence of mutations was higher in the advanced phase of the disease (AP and BC) than in the CP (p = 0.0360), indicating an increase in genomic instability as the disease progresses (Table 2). Patients with high Sokal (p = 0.003), Hasford (p = 0.008), and EUTOS (p = 0.0002) score showed significantly higher incidence of mutations, as compared to patients with low/intermediate score.

### Mutations in the BCR/ABL kinase domain

**Type and location of mutation**: Among the 94 imatinib resistant CML patients, 37 (39%) patients had mutations in the ABL kinase domain of the BCR/ABL gene. The frequency of mutations was higher in the P loop accounting to 43% of the total mutations in the kinase domain. The most common and highly resistant mutation in the P loop was Y253H/F with a frequency of 22% (8/37) and the PPhV2 damaging score of 1, followed by G250E 11% (4/37) and with the damaging score of 0.993. The other point mutations were M244V, K247E and E255V, with the frequency of 3% (1/37) each and a deletion mutation L248-K274 was also found in the P-loop. In the gatekeeper region T315I and F317L occupied 14% (5/37), whereas the activation region (L387M, H39R) occupied 8% (3/37) of the total mutations. Two insertion mutations were detected in the catalytic region of the ABL kinase domain, along with 5 point mutations. One was in-frame tri-nucleotide (GAA) insertion mutation detected at the 357^th^ amino acid position of the catalytic region in one patient (reported previously) and another was the insertion of 35bp of intron 8 at the exon 8–9 junction, detected in 3 patients. The other point mutations found were M351T, M351V, E355G, F359V and F359I. Only two patients were found to have multiple mutations.

**Fig 1:**
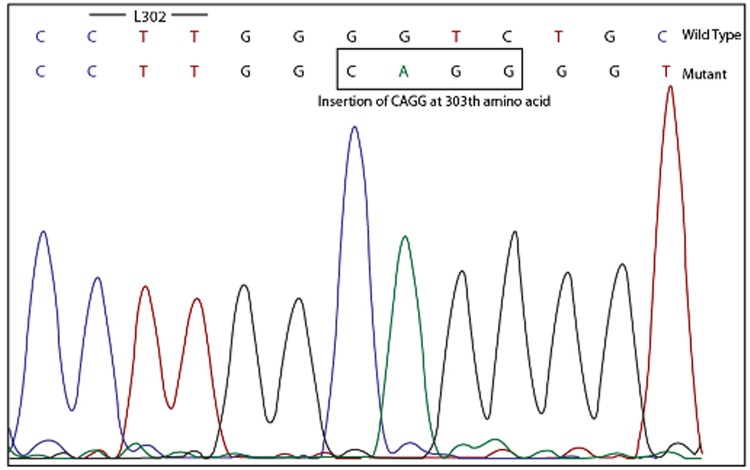
Electropherogram showing the insertion mutation ΔCAGG at 303^th^ amino acid position between P-loop and gatekeeper region.

**Novel mutations**: In this study, a novel insertion mutation, insΔCAGG at 303^th^ amino acid position between P-loop and gatekeeper region was identified (Fig. 1). The second novel mutation found in the kinase domain was the point mutation F493L, located below the activation loop. This mutation was present in the same patient having insertion CAGG at 303^th^ amino acid position. The damaging score calculated for this mutation using PolyPhen2 software predicted it to be benign, giving it a score of 0.102

### Mutations in the SH3-SH2 domain of the BCR/ABL

The regulatory domain of the BCR/ABL fusion gene includes the cap region, the SH3 region, the SH2 region and the linker between the two. Four (4) of 94 (4%) patients showed the presence of mutations in the regulatory domain of the BCR/ABL gene. Two novel point mutations, K84E and Y167H, in two different patients were detected in the regulatory regions whereas, the other two patients exhibited the presence of deletion of exon 4.To assess whether the novel point mutation was a polymorphism we sequenced 30 control samples. However, no change was observed at the locus. Thus, confirming that the mutation was novel.

### Haematologic and cytogenetic response of IM resistant CML patients at the time of mutation analysis

Table 3 shows the distribution of the patients according to their haematologic and cytogenetic response at the time of disease resistance. The frequency of mutations was higher in patients with haematological resistance than the cytogenetic resistance, 62% and 43% respectively (p = 0.0346).

**Table 3:** Distribution of the patients according to their haematologic and cytogenetic response at the time of disease resistance.

	CHR	PHR	No HR	CCyR	Partial CyR	Minor CyR	Minimal CyR	No CyR	Total
	n/N(%)	n/N(%)	n/N(%)	n/N(%)	n/N(%)	n/N(%)	n/N(%)	n/N(%)	
**CP**	9/46	16/23	4/13	1/3	4/29	7/18	9/16	8/10	29/76
**AP**	1/4	2/2	2/3	0/0	0/2	1/2	2/2	2/3	5/9
**BC**	0/0	1/2	6/7	0/0	2/2	1/1	1/1	3/5	7/9

Frequency of mutations in patients with Haematologic resistance: 31/50 (62%)

Frequency of mutations in patients with cytogenetic resistance: 39/91 (43%)

CP: Chronic Phase

AP: Accelerated Phase

BC: Blast Crisis

CHR: complete Haematologic Response

CCyR: Complete Cytogenetic Response

n: Number of patients with mutations

N: Number of imatinib resistant patients

### Molecular monitoring as trigger for mutations

All patients (n = 245) in a complete haematologic and complete cytogenetic response were monitored closely for the BCR/ABL transcript levels. Eighty five patients with an initial complete haematologic and complete cytogenetic response exhibited consecutive increase in transcript level of at least 1logat three different occasions and were considered for mutation analysis. Among the patients with an increase in transcript level of more than 2 log,64% (29/45) of the patients had mutations, whereas 20% (8/40) patients with more than 1 log increase in transcript level had mutations. Thus a 2 log rise in transcript level strongly indicated the presence of mutation (p = 0.001).

### Impact of non-TKI and TKI treatment on first mutations

**Fig 2:**
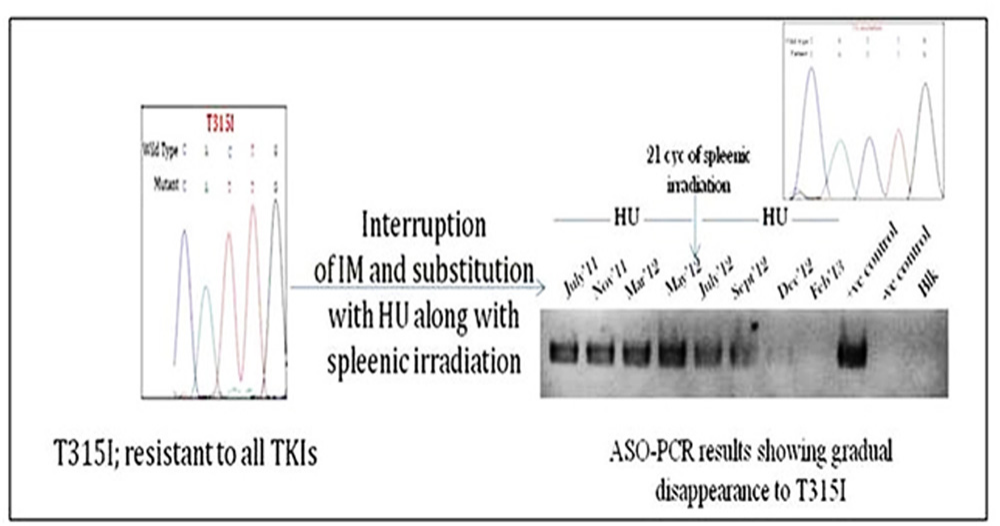
Diagram illustrating gradual disappearance of T315I mutation with hydroxyurea and spleenic irradiation treatment.

All the patients with mutations in the ABL KD, except for patients with T315I, F317L and M351T/V, were put on dasatinib. Majority of the patients were benefited by the dasatinib treatment without selection of a second mutation. A patient with F317L was put on 600mg/day nilotinib after this mutation was detected. After 8 months of nilotinib treatment, mutation analysis of BCR/ABL KD revealed selection of a second mutation E255V along with the first mutation F317L. All the four patients with T315I mutation were put on hydroxyurea. Of the four, one exhibited a huge spleen along with TLC of 1.64L. In view of massive splenomegaly, the patient was given palliative spleenic irradiation along with hydroxyurea for cytoreduction. Interestingly, this treatment resulted in a gradual disappearance of T315I mutation along with a sharp drop in the total count. The gradual disappearance of T315I mutation was confirmed with ASO-PCR specific for T315 and I315 mutation (Fig. 2).

### Non-TKI pre-treatment and occurrence of mutations

**Fig 3:**
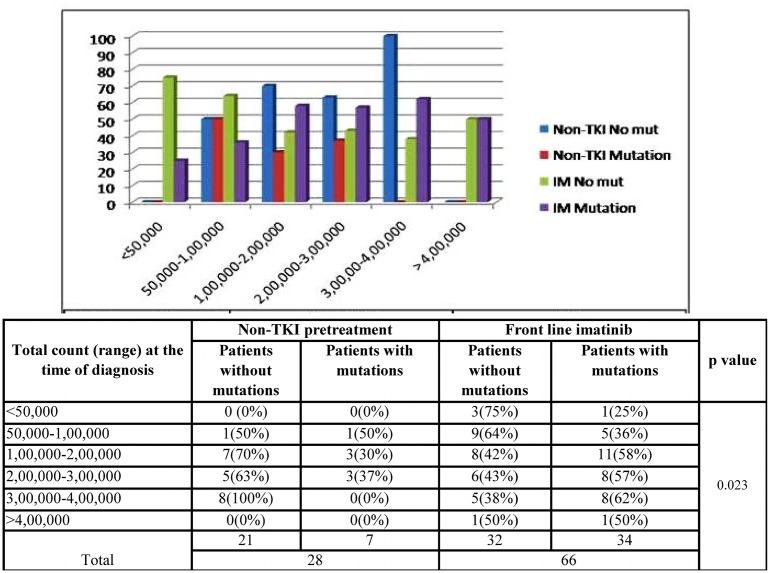
Bar graph shows the distribution of IM resistant CML patients according to the first line treatment and the total count at the time of diagnosis. As the WBC count increases, the difference between the lengths of the blue and the red bars increases to a greater extent as compared to the difference in lengths between the green and violet bars, indicating less number of mutations in patients pre-treated with a non-TKI (HU) as compared to the patients with first line imatinib.

Among the 28 imatinib resistant CML patients pre-treated with a non-TKI (HU), mutations were detected in only 7 patients whereas, 34 patients had mutations in the BCR/ABL gene among the 66 imatinib resistant patients with front-line imatinib. According to the graph (Fig. 3), patients with high counts (>1,00,000/μl) were benefited by a non-TKI pre-treatment. At high counts, less number of mutations were detected in patients pre-treated with a non-TKI as compared to the patients with front line imatinib (p = 0.023). Also, the median duration of occurrence of mutation was noted to be 41 months for patients with first line imatinib and 85 months for the patients pre-treated with hydroxyurea.

### Survival analysis of patients with mutation according to the disease phase

**Fig 4:**
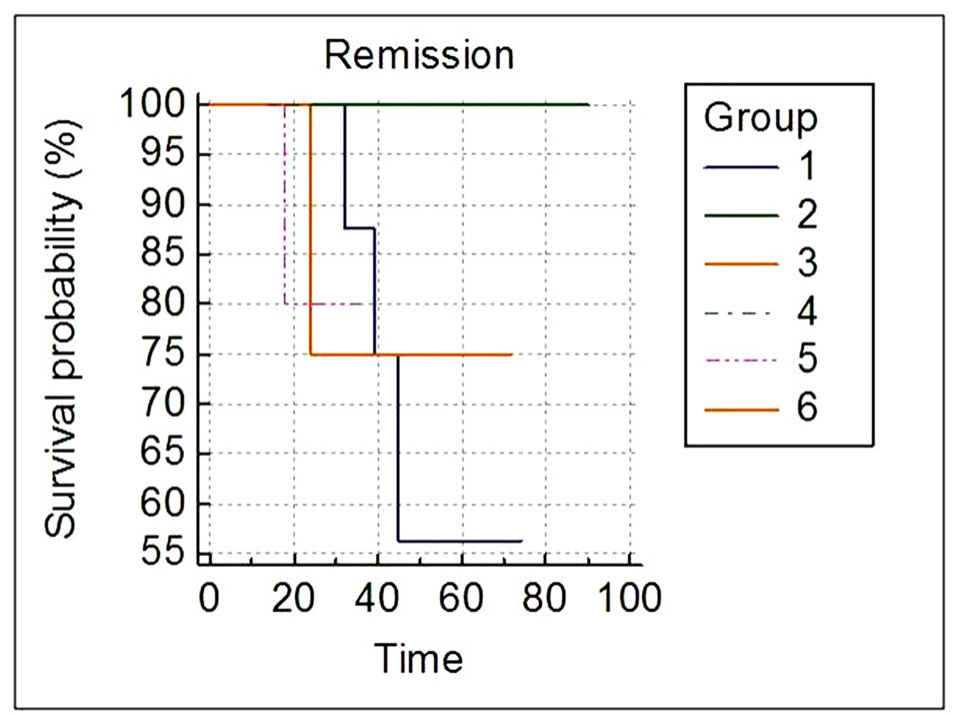
Kaplan-Meier survival curves according to the mutations and the disease phase. Group 1: Chronic Phase (P-Loop); Group 2: Chronic Phase (non P-Loop); Group 3: Accelerated Phase (P-Loop); Group 4: Accelerated Phase (non P-Loop); Group 5: Blast Crisis (P-Loop); Group 6: Blast Crisis (non P-Loop).

Survival analysis was done to investigate if the outcome of mutations depend on the phase of the disease. Patients were divided into two groups depending upon the out-come of mutations to imatinib; P-loop + gatekeeper (here termed as P-loop) and non P-loop. Significantly different overall survival was observed (p = 0.0448) for the P-loop and non P-loop mutations in the chronic phase of the disease, whereas the difference was not significant for the two groups of mutations in AP (p = 0.6171) or BC (p = 0.6547) (Fig. 4).

### Duplication of Ph chromosome

Of the 94 CML patients showing resistance to imatinib, only 1 patient showed duplication of Philadelphia. At the time of diagnosis, FISH analysis and conventional karyotyping in this patient revealed single Philadelphia chromosome with 9qdel and 22qdel. At the time of resistance, FISH analysis and conventional karyotyping in the same patient revealed duplication of Philadelphia chromosome along with i(17q) (figure not shown).

## Discussion

Of the total 254 CML patients included in the study 94 (37%) failed to respond to the imatinib treatment. In comparison to the western population the response rate in our study was found to be very poor. The factors contributing to the low response rate could be attributed to the poor hygiene, does not militating proper compliance.

BCR/ABL mutations are responsible for a substantial proportion of imatinib resistance. In our study, the entire BCR/ABL gene was sequenced including the SH3-SH2 domain, cap region and the ABL kinase domain and the overall incidence of mutations in 94 CML patients with imatinib resistance was 44% (41/94), at the median of 40 months (range 3–96 months). The incidence was similar to those reported in other studies [5, 8, 9]. The frequency of mutations was more in the advanced phase of the disease than in the chronic phase (p = 0.0360). Previous studies to detect mutation rates on tumour-cell lines have found that the mutation rates are roughly equal to those in normal cell lines, with the exception of tumour lines with reported defects in mismatch repair (MMR) or nucleotide-excision repair (NER) [10, 11]. This gives us a hint of the possible role played by defective MMR and NER pathways in the advanced stage of CML. Similar to other studies [12, 9], in this study more patients with haematologic resistance had mutations compared to patients with cytogenetic resistance (62% v/s 43%, p = 0.0346). Qin et al reported that 70% of patients with haematologic resistance had mutations as compared to 44% of patients with cytogenetic resistance [12]. Also, on the basis of molecular monitoring our study suggested that an increase in transcript level of more than 2 log strongly suggested presence of mutation. However, a mutation is typically present several months before an increase in BCR-ABL levels [13] and hence molecular analysis data should not be considered as a standard to trigger mutation analysis, so as to avoid delay in mutation detection.

Among the 94 imatinib resistant patients, 39% had mutations in the ABL kinase domain whereas just 4% had mutations in the SH3-SH2 domain. The K84E mutation in the SH3-SH2 domain was found to be highly resistant to imatinib, clinically and on PolyPhen 2 scale as well. In case of Y167H, the substitution was found to be less damaging on PolyPhen 2 scale and also clinically and was overcome by dosage adjustment. Majority of the mutations were clustered in the P-loop, accounting to 43% of the total BCR/ABL gene mutations. Y253H/F was the most common mutation with a frequency of 22% (8/37). In our study, the occurrence of this mutation was observed to be associated with advanced disease phase (1 in CP v/s 7 in AP and BC). Members of the Src family of kinases which have increased kinase activity relative to ABL, all have a phenylalanine at amino acid position 5 in the P loop, whereas ABL has a tyrosine at this position [14]. Substitution of tyrosine by phenylalanine at Y253^th^ position might result in an increased kinase activity. Furthermore, the Y253F mutation, when engineered into ABL has demonstrated onco-genic activity [15]. These data suggest the possible role played by Y253H/F in increasing the genetic instability and driving the disease to a more advanced phase. However, Y253H/F mutation is the cause for or the result of the advanced phase remains unanswered. This study identified 3 insertion/deletion mutations; 2 insertion and 1 deletion. One was the novel in-frame tri-nucleotide (GAA) insertion mutation detected at the 357^th^ amino acid position of the catalytic region, which we have reported [16]. The second was a deletion mutation ΔL248-K274 in the P-loop. To test the effect of this deletion mutation, Sherbenou et al [17] grew the ΔL248-K274 mutant BaF3-p210 cell line in absence of IL-3. Unexpectedly, co-expression led to an increase in imatinib sensitivity. Furthermore, the increase in sensitivity was consistently highest in the lines expressing the highest level of deletion mutant. Considering the fact that the P-loop is involved in ATP-binding, its deletion might have rendered the protein non-functional. This observation indicates that there is a dominant-negative effect of deletion mutant with respect to imatinib sensitivity. In our case, a mosaic pattern was observed, indicating the presence of the deletion mutant along with the wild type BCR/ABL. Since no other mutation was observed in this patient, it can be supposed that the resistance to imatinib might be due to some other mechanism and not due the deletion mutation. The third mutation found was the novel mutation, insertion of CAGG at 303^th^ amino acid position between P-loop and gatekeeper region. Since the position of the mutation is critical, it might have disrupted the imatinib binding site and led to resistance.

CML with T315I mutation has been reported to have poor prognosis [18], owing to its obstinate resistance to all the first and second generation TKIs. All the four patients in our study with this mutation were put on hydroxyurea. Of these four, only one showed evidence of massive splenomegaly and was given a palliative spleenic irradiation along with hydroxyurea, which resulted in gradual disappearance of T315I along with drop in total count, apparently resulting in a temporary response. Since the leukemic stem cell bears the mutation and both the therapies used here have their activities against the leukemic stem cells, this has left us clueless as to which therapy actually led to the disappearance of this mutation. But as per our experience, the other three patients with this mutation and on HU therapy didn’t show disappearance of this mutation. Moreover, the patient consistently exhibited this mutation on HU for nearly 10 months and then started exhibiting a gradual disappearance of this mutation after spleenic irradiation. We hypothesise that spleenic irradiation might have played a role in disappearance of this mutation, since spleenic microenvironment is known to alter the biology of spleenic leukemic stem cells. Thus further studies on the role of HU or spleenic microenvironment on leukemic stem cells bearing the mutation may be worth considering.

In our study, the survival analysis revealed that the disease phase at the time of resistance may not be a deciding factor for the clinical outcome with context to the mutations. The influence of mutations on the clinical outcome in AP and BC was observed to be insignificant, indicating the active involvement of more potent drug resistance mechanisms in AP and BC. The influence of P-loop mutations was seen to be more pronounced over non P-loop in chronic phase. Some of the reasons for this might be the less number of genetic lesions and more number of terminally differentiated cells in chronic phase. In contrast to our study, Kim et al [8] reported a marked influence of P-loop mutation over non P-loop mutations in BC.

Pre-treatment with non-TKIs has always been a topic of controversy. Many studies have reported that front-line imatinib treatment should be considered as the first choice of treatment. However, here we present a slightly different view. In our study, the median duration of occurrence of mutation was noted to be 41 months for patients with first line imatinib and 85 months for the patients pre-treated with hydroxyurea or busulfan. This might be more likely due to the cytoreduction by the cytotoxic drug, hydroxyurea, reducing the burden on the subsequent TKI therapy and leading to a more effective response. Another reason might be the enhanced effect of non-specific therapy on more primitive cells or leukemic stem cells, in contrast to the currently approved TKIs, which are probably not able to eradicate the rare leukemic population [19]. Also, the cytotoxic treatment would have targeted the CML stem cells which are not’BCR/ABL oncogene addicted’ and are considered to be the contributors of subsequent resistance. These results suggest that, an initial non-TKI pre-treatment prolongs the period without detection of BCR/ABL mutations and are also in concordant with the concept that mutants expand under the selective pressure of imatinib. These data also suggest that depleting stem cell pool by non-TKI pre-treatment would leave fewer stem cells available to develop mutations, thereby taking away some of the targets of resistance. Our data supports the finding of Razqa et al. 2012 [19], that who stated that pretreatment with non-specific non-TKI drugs prior to TKI therapy does not preferentially select for initial BCR-ABL1 KD mutations, in contrast to first-line imatinib therapy. Also, depletion of stem cell pool could also be one of the reasons of why some imatinib discontinuation studies [20, 21, 22] have lower relapse rates in patients receiving prior hydroxyurea or interferon treatment. Though the initial cytotoxic pre-treatment prolongs the period without detection of BCR/ABL mutations, the duration of pre-treatment should be considered as a crucial factor for the reason that prolonged treatment with hydroxyurea may contribute to DNA damage and mutations.

## Conclusion

Results of our study showed that among the different BCR/ABL dependent mechanisms of imatinib resistance, kinase domain mutations of the BCR/ABL gene are more prevalent, followed by mutations in the SH3-SH2 domain and then BCR/ABL amplification with the least frequency. In view of the fact, that all the mutations were detected after the start of the therapy, expansion of BCR/ABL gene mutation in CML can be considered to be time dependent and dependent on pressure exerted by tyrosine kinase inhibitor. Our study also revealed a clear impact of non TKI pre-treatment. Hence, close monitoring of CML patients is essential to assist in proper management of the disease.
